# Arbuscular mycorrhizal fungi and *Trichoderma harzianum* alter salicylic acid–jasmonic acid balance to suppress *Fusarium* wilt in tomato

**DOI:** 10.3389/fpls.2025.1714648

**Published:** 2025-11-18

**Authors:** Kangxu Zhang, Haixi Wang, Wei Xie, Tianyi Niu, Wei Fu, Xin Zhang, Zhipeng Hao, Baodong Chen

**Affiliations:** 1 State Key Laboratory of Regional and Urban Ecology, Research Center for Eco-Environmental Sciences, Chinese Academy of Sciences, Beijing, China; 2 University of Chinese Academy of Sciences, Beijing, China; 3 China CAS Key Laboratory of Mountain Ecological Restoration and Bioresource Utilization & Ecological Restoration and Biodiversity Conservation Key Laboratory of Sichuan Province, Chengdu Institute of Biology, Chinese Academy of Sciences, Chengdu, China

**Keywords:** plant disease biocontrol, hormonal balance, arbuscular mycorrhizal fungi, Trichoderma harzianum, jasmonic acid, salicylic acid

## Abstract

*Fusarium* wilt, caused by *Fusarium oxysporum*, poses a significant challenge to tomato production, and sustainable control strategies are urgently needed. Beneficial microbes such as arbuscular mycorrhizal (AM) fungi and *Trichoderma harzianum* are widely applied as biocontrol agents, but their combined effects and the underlying immune mechanisms in host plants remain insufficiently understood. In this study, greenhouse experiments were conducted to evaluate the impacts of inoculation with *Rhizophagus irregularis* and *T. harzianum*, individually and together, on pathogen colonization, nutrient uptake, hormone signaling, and defense responses in tomato. Quantitative PCR revealed that both beneficial fungi significantly reduced *F. oxysporum* colonization in roots, yet co-inoculation did not provide additional suppression compared with single inoculations. Hormonal profiling showed that pathogen infection alone activated jasmonic acid (JA)-dominated defenses, whereas inoculation with either AM fungi or *T. harzianum* redirected immunity toward a salicylic acid (SA)-associated state. This shift was characterized by elevated SA accumulation, increased activity of phenylalanine ammonia-lyase (PAL) and polyphenol oxidase (PPO), and reduced levels of JA and its derivatives. Dual inoculation reproduced these hormonal and enzymatic changes but did not further enhance them. Correlation analysis revealed that SA enrichment and PAL/PPO activities were negatively associated with pathogen abundance, whereas JA-related compounds correlated positively with disease severity. These findings suggest that beneficial fungi mitigate *Fusarium* wilt by reprogramming host immune responses from JA- to SA-dominated pathways, but their combined application does not produce additive benefits. This work provides new insights into the hormonal trade-offs underlying microbe-induced resistance and informs the design of microbial consortia for sustainable plant disease management.

## 1 Introduction

Plants coexist with diverse soil microorganisms that strongly influence their ability to withstand pathogen attack (Zhang et al., 2025). In response to biotic stresses, they have evolved sophisticated immune strategies to detect pathogens and activate defense responses (Deng and He, 2024). Central to these immune responses are phytohormonal signaling networks (Yang et al., 2015), particularly those mediated by salicylic acid (SA) and jasmonic acid (JA). SA is typically linked to resistance against biotrophic pathogens, while JA is crucial for defending against necrotrophic pathogens and herbivorous organisms (Zust and Agrawal, 2017). Balance between these signaling pathways coordinates the induction of defense-related genes and the activation of protective enzymes such as phenylalanine ammonia lyase (PAL) and polyphenol oxidase (PPO), which together strengthen the plant’s resistance capacity (Robert-Seilaniantz et al., 2011; Hou and Xu, 2025). Although immune activation often comes with metabolic costs, the ability of plants to fine-tune SA–JA interactions allows dynamic adjustment of defense without fully compromising growth (Li et al., 2022). Importantly, accumulating evidence shows that rhizosphere microorganisms can actively shape these signaling networks, modulating hormone profiles and defense gene expression to enhance host resistance.

Tomato *Fusarium* wilt, caused by *Fusarium oxysporum* f. sp. *lycopersici* (Fo), is a destructive soil-borne disease (Buddenhagen, 2009). Fo exhibits diverse transmission routes, and multiple physiological races, making it challenging to control in agricultural systems (Di Pietro et al., 2003). After root penetration, the pathogen colonizes xylem vessels, where abundant conidia, together with secreted extracellular polysaccharides and toxins such as fusaric acid, block water transport, leading to characteristic wilting and eventual plant death (Srinivas et al., 2019). Yield losses range from 20–30% in infected fields to over 80% in severe outbreaks, posing a significant bottleneck for tomato cultivation (Gordon, 2017).

Arbuscular mycorrhizal (AM) fungi are obligate biotrophic organisms that form mutualistic relationships with approximately 80% of land plant species (Shi et al., 2023). Both fossil records and molecular data indicate that this symbiotic association emerged over 400 million years ago. In this mutualism, plants allocate photosynthetic carbon to AM fungi, which enhance nutrient uptake (e.g., phosphorus) via extraradical hyphae (Kiers et al., 2011). AM fungi are often associated with enhanced plant resistance against diverse pathogens, including fungi, bacteria, nematodes, and viruses, although outcomes may vary depending on host–pathogen–fungus interactions (Hao et al., 2019; Wang et al., 2022). For instance, AM fungi inoculation has been shown to significantly reduce the incidence of cucumber *Fusarium* wilt (Ahammed et al., 2020). AM fungi suppress plant diseases through various mechanisms, including competition for infection sites and nutrients, and activation of the plant’s immune system (Weng et al., 2022). This enhanced defense response, often mediated by JA signaling, helps plants resist pathogen invasion, thereby reducing disease severity and improving overall plant health (Zhang et al., 2019).

*Trichoderma*, a genus of beneficial fungi, is widely used to control soil-borne pathogens (Dutta et al., 2023). Approximately 60% of fungal-based biopesticides globally contain *Trichoderma* strains (Iqbal et al., 2024). These fungi antagonize pathogens through mycoparasitism, the biosynthesis of bioactive metabolites like plant growth regulators, enzymes, siderophores, antibiotics, secondary metabolites, and niche competition (Mukherjee et al., 2012). *T. harzianum* isolates have shown antagonistic effects against *F. oxysporum*, inhibiting mycelial growth and mitigating plant damage (Chohan et al., 2024). Both AM fungi and *Trichoderma* are well-established biocontrol agents, yet their combined effects remain contentious.

Some studies report synergistic suppression of *Fusarium* wilt in crops like soybean, where AM fungi-*Trichoderma* consortia enhance nutrient uptake and photosynthetic efficiency (Eke et al., 2019; Hewedy et al., 2020). Conversely, no additive benefits were observed in banana and melon systems, indicating strain- or host-specific interactions (Castillo et al., 2019). Likewise, in onion, applying AM fungi alone outperformed its combination with *T. harzianum* (Yağmur et al., 2024). This lack of synergy may result from differing mechanisms of action, where the beneficial effects of each microorganism may overlap or interfere with one another in certain host plants, reducing the potential for enhanced disease suppression (De Jaeger et al., 2011; Thakur et al., 2019). Additionally, host plants may preferentially activate one defense pathway over another, limiting the overall effectiveness of combined inoculations.

AM fungi and *Trichoderma* enhance plant disease resistance by modulating the host immune system (Salwan et al., 2022). In mycorrhizal plants, AM fungi induce mycorrhiza-induced resistance (MIR), a defense mechanism characterized by complex signal transduction and physiological processes that enhance plant immunity (Cameron et al., 2013). Similarly, *Trichoderma* suppresses pathogen invasion by modulating the SA signaling pathway, thereby triggering systemic disease resistance in host plants (Grant and Lamb, 2006). MIR is considered a form of induced systemic resistance (ISR), typically mediated by JA signaling pathways in response to rhizosphere microbes or biocontrol agents (Pieterse et al., 2009). In contrast, systemic acquired resistance (SAR), a pathogen-triggered defense response, primarily relies on the SA signaling pathway. Current models suggest that JA confers resistance against necrotrophic pathogens, while SA is critical for combating biotrophic pathogens, often creating an antagonistic trade-off between these defense pathways (Lorenzo and Solano, 2005). However, *F. oxysporum*, a hemibiotrophic pathogen, presents unique challenges due to its complex infection dynamics, making the interplay of defense pathways under dual inoculation with functional microbes like AM fungi and *Trichoderma* still unclear.

The biocontrol potential of AM fungi and *Trichoderma* has been well-documented, yet their combined effects on *F. oxysporum* colonization and the underlying immune mechanisms in tomato remain insufficiently explored. This study aims to evaluate the impact of AM fungi and *Trichoderma* inoculation on *F. oxysporum* colonization in tomato roots using greenhouse pot experiments that simulate natural wilt disease conditions. Additionally, we will investigate the defense mechanisms activated in pathogen-infected tomato plants under AM fungi and *Trichoderma* inoculation by measuring the concentrations of key plant hormones and evaluating the expression of defense-related genes and enzymes involved in immune responses. We hypothesize that: (1) Inoculation with AM fungi and *Trichoderma* will reduce *F. oxysporum* colonization in tomato roots through distinct yet complementary mechanisms, with AM fungi primarily enhancing nutrient acquisition and *Trichoderma* directly suppressing pathogen growth. (2) Dual inoculation with AM fungi and *Trichoderma* will synergistically enhance plant resistance to *Fusarium* wilt by fine-tuning the immune response, shifting plant defense from pathogen-driven mechanisms to microbially-induced resistance. By integrating these approaches, this study aims to elucidate the defense mechanisms activated under the co-inoculation of beneficial fungi and provide practical insights for optimizing microbial consortia in disease management.

## 2 Materials and methods

### 2.1 Plant material and seed preparation

Tomato (*Lycopersicon esculentum* Mill.) cultivar ‘Shouhe’ was provided by Shouguang Xinxinran Horticulture Co., Ltd., Shandong, China. The seeds were sterilized on the surface using a 2% (v/v) sodium hypochlorite solution (available chlorine 5.2%, Sinopharm Chemical Reagent Co., Ltd., Shanghai, China) for 10 minutes, followed by five rinses with sterile deionized water. Afterward, they were placed on damp filter paper and incubated in the dark at 25 ± 1°C for 48 hours to allow germination.

### 2.2 Soil characteristics and substrate preparation

Soil samples were collected from the Yanqing Field Experiment and Demonstration Station (40°47′N, 116°34′E) in Beijing, China, which is affiliated with the Research Center for Eco-Environmental Sciences, Chinese Academy of Sciences. The soil was a sandy loam and exhibited the following physicochemical properties: organic matter, 44.21 g kg^-^¹; NO_3_^-^-N, 8.28 mg kg^-^¹; NH_4_^+^-N, 5.54 mg kg^-^¹; total phosphorus, 0.87 g kg^-^¹; available phosphorus (Olsen-P), 15.01 mg kg^-^¹; available potassium, 204.06 mg kg^-^¹; and pH (1:2.5, w/v), 7.68. The soil was passed through a 2 mm sieve and sterilized using γ-radiation at a dose of 20 kGy. Basal fertilizers were applied at 120 mg N kg^-^¹, and 20 mg P kg^-^¹ according to Xie et al. (2019).

### 2.3 Microbial strains and culture conditions

The AM fungal strain *Rhizophagus irregularis* CGMCC12157 (Ri) used in this study was originally isolated from rhizosphere soil collected in Anhui Province, China. The strain has been stored in the China General Microbiological Culture Collection Center (CGMCC), and is also maintained at the Bank of Glomeromycota in China (BGC), Beijing, China. The Ri inoculum was composed of a sterilized mixture of zeolite, sand, and sandy loam soil, enriched with colonized *Sorghum bicolor* root fragments, extraradical hyphae, and spores at a concentration of 42 spores g^-^¹.

*T. harzianum* CFCC82908 (Th) was sourced from the China Forestry Culture Collection Center (CFCC) in Beijing, China. Initially, the fungus was cultured on potato dextrose agar (PDA) at 25°C for 7 days. Afterward, hyphal plugs were transferred to 100 mL of potato dextrose broth (PDB) and incubated at 25°C with agitation at 120 rpm for another 7 days. The resulting suspension was passed through a triple-layer sterile gauze, followed by centrifugation at 3000 × g for 5 min (performed twice). It was then washed three times with sterile deionized water and resuspended. The concentration was ultimately set to 3 × 10^6^ conidia mL^-^¹ determined using a hemocytometer (El-Sharkawy et al., 2018).

*F. oxysporum* f. sp. *lycopersici* (Fo) was kindly provided by Prof. Yingzi Yun from Fujian Agriculture and Forestry University. Prior to use, the strain was authenticated based on morphological and molecular identification and confirmed for pathogenicity on tomato plants. The isolate was maintained on PDA at 25°C for 7 days, then subcultured into 100 mL PDB and incubated under the same conditions as Th. Conidia were harvested following the same filtration, centrifugation, and washing steps as for *T. harzianum*. The concentration was adjusted to 1 × 10^8^ conidia mL^-^¹ (Ouyang et al., 2014).

### 2.4 Experimental design and cultivation conditions

A full factorial design was employed to investigate the effects of three microbial factors: *R. irregularis* (Ri), *T. harzianum* (Th), and *F. oxysporum* (Fo). Each factor had two levels: presence (+) and absence (−), resulting in 8 treatment combinations. Each treatment included 9 replicates, totaling 72 pots arranged in a randomized complete block design. Blocks were spatially assigned within the greenhouse to minimize environmental variation.

Each pot (15 cm diameter × 12.5 cm height) was filled with 700g of sterilized soil. In +Ri treatments, a 300 g of soil was thoroughly mixed with 50 g of AM inoculum (~2,100 spores) and layered centrally within the pot. For −Ri treatments, an equivalent amount of autoclaved inoculum was used, supplemented with 5 mL of a microbial filtrate (<20 μm) prepared by suspending live AM inoculum in sterile water (1:4 w/w) and filtering through a nylon mesh. This filtrate was added to restore the native non-mycorrhizal microbial community (Hao et al., 2012). The effectiveness of Ri inoculation was validated through trypan blue staining (Biermann and Linderman, 1981). Microscopic analysis revealed that roots from inoculated treatments exhibited a colonization rate exceeding 34%, whereas no mycorrhizal structures were observed in roots from non-inoculated controls.

The inoculation of Th was performed 7 days post-transplantation by applying 40 mL of a conidial suspension (3 × 10^6^ conidia mL^-^¹) to the soil near the root zone. This staggered inoculation strategy was employed to minimize interference during the early stages of AM fungi colonization, which requires several days for hyphal establishment and arbuscule formation. Delayed inoculation of *Trichoderma* thus offers a temporal niche separation, potentially enhancing co-beneficial effects (Tanwar et al., 2013). The presence of Th following inoculation was verified by plating root-associated samples on *Trichoderma* selective medium (TSM), a medium specifically formulated to promote the growth of *Trichoderma* spp. while inhibiting non-target fungi (Elad and Chet, 1983).

Inoculation with Fo was carried out 4 weeks after transplantation, a stage at which the tomato root system was sufficiently developed to allow consistent pathogen colonization and symptom expression. This timing also ensured that prior microbial inoculants had established effective interactions with the host root system, thereby enabling an accurate assessment of biocontrol effects (Wang et al., 2022). To facilitate infection, tomato roots were gently wounded using a sterile scalpel, and 4 mL of Fo conidial suspension (1 × 10^8^ conidia mL^-^¹) was applied directly to the injury sites, following the method of Ouyang et al. (2014).

For all −Th and −Fo control treatments, the same procedure was followed using an equal volume of sterile distilled water to ensure consistency in mechanical disturbance and moisture application.

Tomato seeds with uniform germination were selected and sown into pots. Two weeks after planting, seedlings were pruned to leave 2 evenly growing plants per pot. The plants were grown in a greenhouse at the Chinese Academy of Agricultural Sciences, under natural light conditions. Day and night temperatures were maintained at 28–30°C and 15–17°C, respectively. Soil moisture was kept at approximately 17% (w/w), equivalent to 75% of field capacity, through daily irrigation with deionized water. A phosphorus-free Hoagland nutrient solution was applied weekly throughout the growth period.

### 2.5 Harvest, biomass measurement, and sample allocation

Plants were harvested 80 days after inoculation with Fo, corresponding to the late infection stage when both *R. irregularis* colonization and *F. oxysporum* establishment reached stable levels. This endpoint was selected to capture steady-state physiological and molecular responses to microbial interactions, rather than transient early-stage fluctuations. Photosynthetic parameters of tomato leaves were measured two days before harvest using a portable photosynthesis system (Li-6800, Li-COR Biosciences, USA) on the third fully expanded leaf from the top of each plant. At harvest, both plants in each pot were collected and considered as a single biological replicate. Shoots and roots were separated at the soil surface and processed immediately. The roots were carefully rinsed with deionized water and dried using absorbent paper. The fresh weights (FW) of both shoots and roots were measured individually. Four 0.5 g samples of root tissue were each homogenized in liquid nitrogen, quickly frozen, and stored at −80°C for later use in DNA extraction, RNA isolation, phytohormone analysis, and enzyme activity assays. The remaining shoot and root tissues were first dried in an oven at 105°C for 30 minutes to inactivate enzymes, then further dried at 70°C for 24 hours until a constant weight was achieved. The dry weight (DW) was measured, and the dried material was finely ground using a stainless steel mill.

### 2.6 qPCR-based quantification of *Fusarium oxysporum* using the *pgx4* gene

Pathogen load in root tissues, quantified by qPCR of the *pgx4* gene, was used as a proxy for disease severity (Hirano and Arie, 2006; Zhou et al., 2022; Li et al., 2025). Root DNA from tomato plants was isolated with the FastDNA^®^ SPIN Kit for Soil (MP Biomedicals, USA), following the manufacturer’s guidelines. The *pgx4* gene was quantified via quantitative PCR (qPCR) using a Bio-Rad CFX96 Real-Time PCR System and SYBR Premix Ex Taq (Takara Biotechnology Co., Ltd., Japan). The primers were *sprl*-f (5’-GATGGTGGAACGGTATGACC-3’) and *sp*rl-r (5’-CCATCACACAAGAACACAGGA-3’) (Hirano and Arie, 2006). The qPCR protocol began with an initial denaturation step at 95°C for 45 seconds, followed by 35 cycles of 95°C for 30 seconds, 64°C for 30 seconds, and 72°C for 1 minute. A melting curve analysis was performed, starting at 70°C for 10 seconds and gradually increasing to 95°C with a ramp rate of 0.5°C s^-^¹. A plasmid containing the *pgx4* amplicon (Sangon Biotech Co., Ltd., Shanghai, China) was serially diluted (10^-^¹–10^-7^) to generate a standard curve. Gene copy numbers were determined from the standard curve based on the DNA concentration, amplicon length, and the average molecular weight of nucleotide base pairs (660 g mol^-^¹ bp^-^¹).

### 2.7 Elemental analysis

Approximately 0.2 g of dried and finely ground shoot or root tissue was digested with 5 mL of concentrated nitric acid (HNO_3_, analytical grade) using a microwave digestion system (MARS 5, CEM Corporation, Matthews, NC, USA). Following digestion, the solution was allowed to cool to room temperature and then diluted to a final volume of 50 mL using ultrapure water in acid-cleaned volumetric flasks. Phosphorus (P) concentration in each sample was measured using inductively coupled plasma optical emission spectrometry (ICP-OES; Prodigy, Teledyne Leeman Labs, Hudson, USA) at a wavelength of 213.617 nm. Calibration was performed using external standards.

### 2.8 Phytohormone analysis

Concentrations of root phytohormones were measured using liquid chromatography–tandem mass spectrometry (LC-MS/MS). About 50 mg of root tissue was rapidly frozen in liquid nitrogen, pulverized into a fine powder, and extracted with 80% methanol supplemented with internal standards. The hormone extracts were filtered and analyzed by LC-ESI-MS/MS using a Shim-pack UFLC SHIMADZU CBM30A system, connected to an Applied Biosystems 6500 triple quadrupole mass spectrometer (Applied Biosystems, Foster City, CA, USA). Chromatographic separation was conducted using a Waters ACQUITY UPLC HSS T3 C18 column (100 mm × 2.1 mm, 1.8 μm particle size). The mobile phase consisted of (A) 0.04% acetic acid in water and (B) 0.04% acetic acid in acetonitrile. The gradient program was as follows: 5% B for 0–1 min, increased linearly to 95% B from 1–8 min, maintained at 95% B for 1 min, then returned to 5% B over 0.1 min and held for re-equilibration until 12 min. Flow rate was 0.35 mL min^-^¹, column temperature was maintained at 40°C, and injection volume was 2 μL. Electrospray ionization (ESI) was performed using both positive and negative ion modes. Source temperature was set to 550°C; ion spray voltage was +5.5 kV in positive mode and −4.5 kV in negative mode; curtain gas pressure was maintained at 35 psi. Hormone concentrations were quantified using an internal standard method with calibration curves generated from authentic reference standards.

### 2.9 Gene expression analysis

Total RNA was isolated from tomato roots with the RNeasy Plant Mini Kit (Qiagen, Germany). First-strand cDNA was synthesized from RNA using the RevertAid First Strand cDNA Synthesis Kit (Thermo Fisher Scientific, USA). Quantitative real-time PCR (qRT-PCR) was conducted with gene-specific primers on a Bio-Rad CFX96 Real-Time PCR Detection System (Bio-Rad, USA). Reactions were carried out in 20 μL of SYBR Green Master Mix (Takara, Japan) with 0.2 μM of each primer and 2 μL of cDNA template. The genes analyzed included *Lipoxygenase D* (*LOXD*, U37840; primers: 5’-ACTCATCAGCACCGACATCG-3’ and 5’-ACTCTCCAGAAAGAACTCCTGC-3’), *Allene Oxide Cyclase* (*AOC*, AW624058; primers: 5’-CTCGGAGATCTTGTCCCCTTT-3’ and 5’-CTCCTTTCTTCTCTTCTTCGTGCT-3’), *PAL* (AW035278 primers: 5’-ACGGGTTGCCATCTAATCTGACA-3’ and 5’-CGAGCAATAAGAAGCCATCGCAAT-3), and *Chitinase* (*CHI*, Z15140; primers: 5’-AACTATGGGCCATGTGGAAGA-3’ and 5’-GGCTTTGGGGATTGAGGAG-3’), with *Ubiquitin-Conjugating Enzyme 3* (*UBI3*, X58253; primers: 5’-TCCATCTCGTGCTCCGTCT-3’ and 5’-GAACCTTTCCAGTGTCATCAACC-3’) serving as the internal reference (Song et al., 2015; Wang et al., 2022). Gene expression levels were determined by the 2^−ΔΔCt^ method (Pfaffl, 2001), normalizing cycle threshold (Ct) values to *UBI3* expression.

### 2.10 Enzyme activity assays

PAL and PPO activities in tomato roots were determined using kits from Solarbio Science and Technology Co., Beijing, China, following the manufacturer’s instructions. Root tissues were blended in extraction buffer and subjected to centrifugation at 12,000 × g for 10 min. The supernatants were used for enzymatic assays. PAL activity was measured based on the conversion of L-phenylalanine to trans-cinnamic acid, recorded at 290 nm, while PPO activity was determined by measuring the increase in absorbance at 410 nm due to quinone formation. Enzyme activities were expressed as units per gram fresh weight (U g^-^¹ FW).

### 2.11 Statistical analysis

Data were examined for normality (Shapiro–Wilk) and homogeneity of variance (Levene), both at a significance level of α = 0.05. To test treatment effects of *R. irregularis* (Myc), *T. harzianum* (Tri), and *F. oxysporum* (Fus) inoculations, a three-factor analysis of variance (ANOVA) was conducted to examine both main and interaction effects. For pairwise comparisons, a one-way ANOVA with Tukey’s honestly significant difference (HSD) *post hoc* test (*p* < 0.05) was applied. Spearman correlation analysis and significance testing were conducted to assess the relationships among the relative abundance of Fo, concentrations of SA, salicylic acid glucoside (SAG), 12-oxo-phytodienoic acid (OPDA), 4-oxo-2-(pentenyl)cyclopentane-1-butanoic acid (OPC-4), JA, jasmonoyl-isoleucine (JA-Ile), methyl jasmonate (MeJA), hydroxylated jasmonic acid (H_2_JA), and jasmonoyl-valine (JA-Val); the expression levels of defense-related genes (*PAL*, *LOX*, *AOC*, and *CHI*); and the activities of PAL and PPO enzymes in tomato roots. Statistical analysis was performed with IBM SPSS Statistics 21.0 (IBM Corp., Armonk, USA), while graphical representations were generated using OriginPro 2021 (OriginLab Corporation, Northampton, USA).

## 3 Results

### 3.1 Pathogen abundance, photosynthesis, plant growth, and phosphorus acquisition

**Figure 1:**
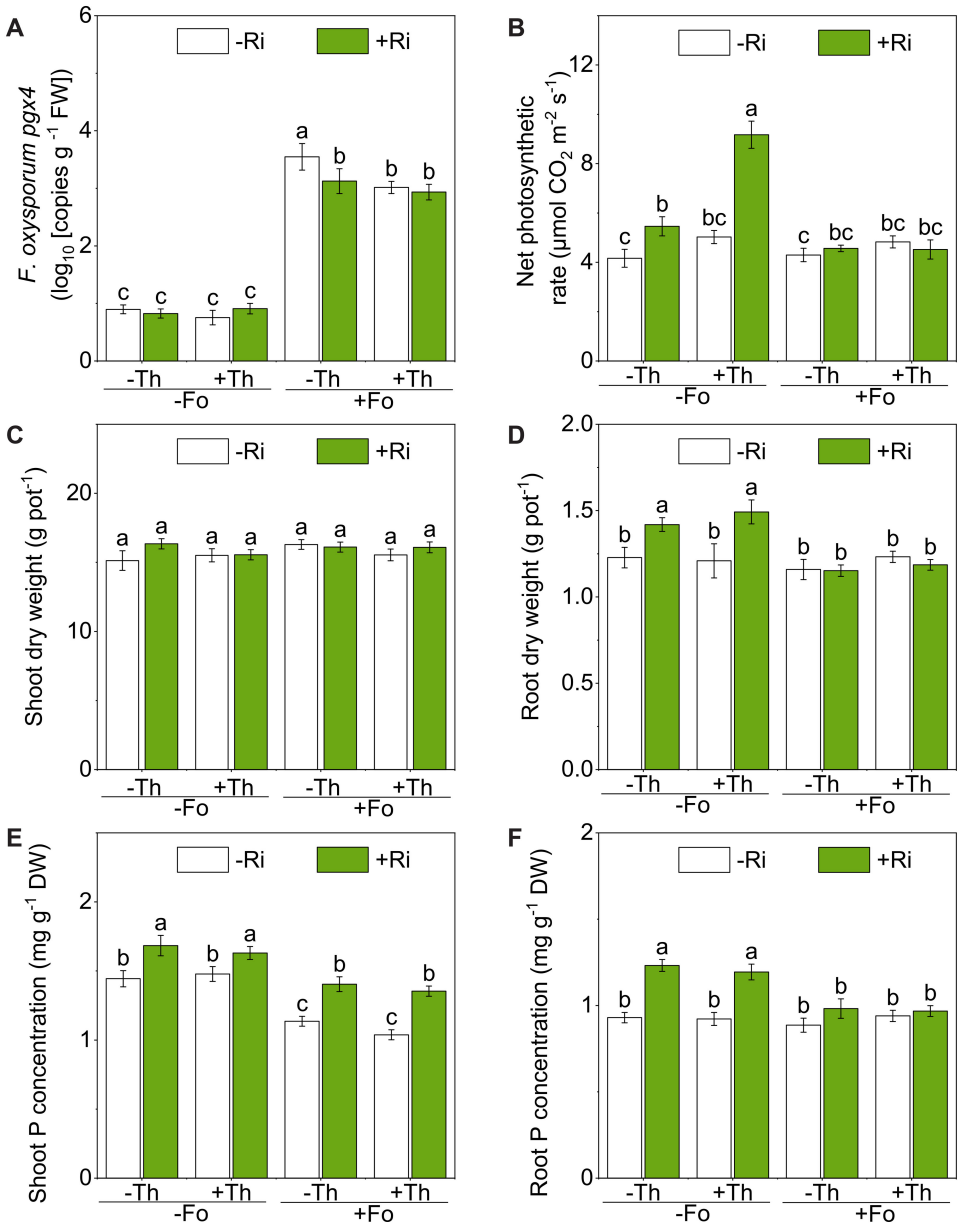
Pathogen abundance **(A)**, net photosynthetic rate **(B)**, shoot dry weight **(C)**, root dry weight **(D)**, shoot phosphorus (P) concentration **(E)**, and root P concentration **(F)** of tomato plants inoculated with/without *Rhizophagus irregularis* (Ri), *Trichoderma harzianum* (Th) and *Fusarium oxysporum* f. sp. *lycopersici* (Fo). The labels -Ri and +Ri represent non-inoculated control and inoculation with Ri, respectively. -Th and +Th represent non-inoculated control and inoculation with Th, respectively. -Fo and +Fo represent non-inoculated control and inoculation with Fo, respectively. The error bars represent the standard error (SE). Columns with the same lowercase letters indicate no significant difference at *p*<0.05.

qPCR targeting the *pgx4* gene confirmed successful colonization of Fo in tomato roots. Plants inoculated with Fo showed significantly higher *pgx4* copy numbers than non-inoculated controls (*p* < 0.001), indicating substantial pathogen accumulation in the root system (**Figure 1A**; **Table 1**). In contrast, inoculation with Ri, Th, or their combination significantly suppressed Fo colonization and proliferation in the root tissues (*p* < 0.05). However, the inhibitory effects of Ri and Th were comparable, and co-inoculation did not yield synergistic suppression of Fo abundance.

**Table 1:** *P*-values from three-way ANOVA showing the effects of experimental factors (*Rhizophagus irregularis*, Myc; *Trichoderma harzianum*, Tri; and *Fusarium oxysporum*, Fus) on a series of dependent variables, including pathogen abundance; net photosynthetic rate (Pn); shoot and root dry weight; shoot and root phosphorus (P) concentrations; phytohormone levels of salicylic acid (SA), salicylic acid 2-O-β-glucoside (SAG), 3-oxo-2-(2-(Z)-pentenyl) cyclopentane-1-butyric acid (OPC-4), 12-oxo-phytodienoic acid (OPDA), jasmonic acid (JA), jasmonoyl-L-isoleucine (JA-Ile), jasmonoyl-valine (JA-Val), methyl jasmonate (MeJA), and dihydrojasmonic acid (H_2_JA); expression levels of lipoxygenase D (*LOXD*), allene oxide cyclase (*AOC*), phenylalanine ammonia-lyase (*PAL*), and chitinase (*CHI*); and the enzymatic activities of phenylalanine ammonia-lyase (PAL) and polyphenol oxidase (PPO).

Three-way ANOVA	Myc	Tri	Fus	Myc × Tri	Myc × Fus	Tri × Fus	Myc × Tri × Fus
log_10_*F. oxysporum pgx4* copies	0.313	0.068	**<0.001**	0.175	0.164	0.116	0.780
Pn	**< 0.001**	**< 0.001**	**<0.001**	**0.029**	**< 0.001**	**< 0.001**	**0.002**
Shoot dry weight	0.205	0.351	0.239	0.730	0.483	0.784	0.135
Root dry weight	**0.012**	0.324	**< 0.001**	0.748	**0.002**	0.748	0.418
Shoot P concentration	**<0.001**	0.252	**<0.001**	0.783	0.192	0.381	0.352
Root P concentration	**<0.001**	0.957	**<0.001**	0.386	**<0.001**	0.446	0.736
SA concentration	0.580	**0.010**	**0.003**	0.431	**0.006**	**0.035**	0.670
SAG concentration	0.370	0.724	0.809	0.887	0.528	0.675	0.804
OPC-4 concentration	0.863	**0.040**	0.072	0.216	**0.041**	0.517	0.294
OPDA concentration	**0.010**	**0.004**	**0.025**	0.941	0.796	0.241	0.896
JA concentration	0.187	**0.018**	0.127	0.080	**0.005**	0.485	0.211
JA-Ile concentration	0.184	**0.012**	0.920	**0.016**	**<0.001**	0.331	0.096
JA-Val concentration	0.521	**0.034**	0.488	**0.039**	0.126	0.735	0.101
MeJA concentration	**0.001**	0.068	**0.004**	0.413	0.502	0.752	0.065
H_2_JA concentration	**0.005**	0.139	0.535	0.413	0.502	0.752	0.065
Relative expression of *LOXD*	**0.015**	0.587	0.889	0.352	0.259	0.080	0.617
Relative expression of *AOC*	0.857	0.104	0.098	0.496	0.956	0.842	0.188
Relative expression of *PAL*	0.237	0.694	**0.022**	0.299	0.453	0.832	0.167
Relative expression of *CHI*	**0.001**	0.870	0.541	0.545	**0.018**	0.853	0.106
Activity of PAL	0.091	0.434	**0.002**	0.191	0.181	0.061	0.120
Activity of PPO	0.984	0.190	**<0.001**	0.948	0.441	0.636	0.096

Bold values indicate statistical significance at *p* < 0.05.

The net photosynthetic rate (Pn) was significantly influenced by Ri, Th, and Fo inoculation, with a notable three-way interaction among these factors (Myc: F(1, 24) = 30.48, *p* < 0.001; Tri: F(1, 24) = 26.71, *p* < 0.001; Fus: F(1, 24) = 32.84, *p* < 0.001; **Figure 1B**; **Table 1**). Under Fo-free conditions, Ri inoculation increased Pn by 31.1% relative to the control, while co-inoculation with Th (Ri + Th) further enhanced Pn by 67.9%.

Microbial treatments did not significantly affect shoot dry weight (**Figure 1C**; **Table 1**). Ri inoculation significantly promoted root dry weight by 15.6% in the absence of pathogen (*p* < 0.05), indicating a classical mycorrhizal growth response (**Figure 1D**; **Table 1**). However, this effect was significantly suppressed under Fo challenge, with a 16.1% reduction in Ri-induced root dry weight, as shown by a significant Myc × Fus interaction (F(1, 64) = 10.60, *p* < 0.01).

Ri enhanced plant P acquisition, increasing shoot and root P concentrations by 16.6% and 32.5%, respectively, compared with uninoculated controls (*p* < 0.001; **Figures 1E, F**; **Table 1**). In the presence of Fo, the Ri-driven increase in root P was reduced by 21.6%, with a significant Myc × Fus interaction detected (*p* < 0.001). Th inoculation had no significant effect on phosphorus acquisition.

### 3.2 Phytohormone profiling

**Figure 2:**
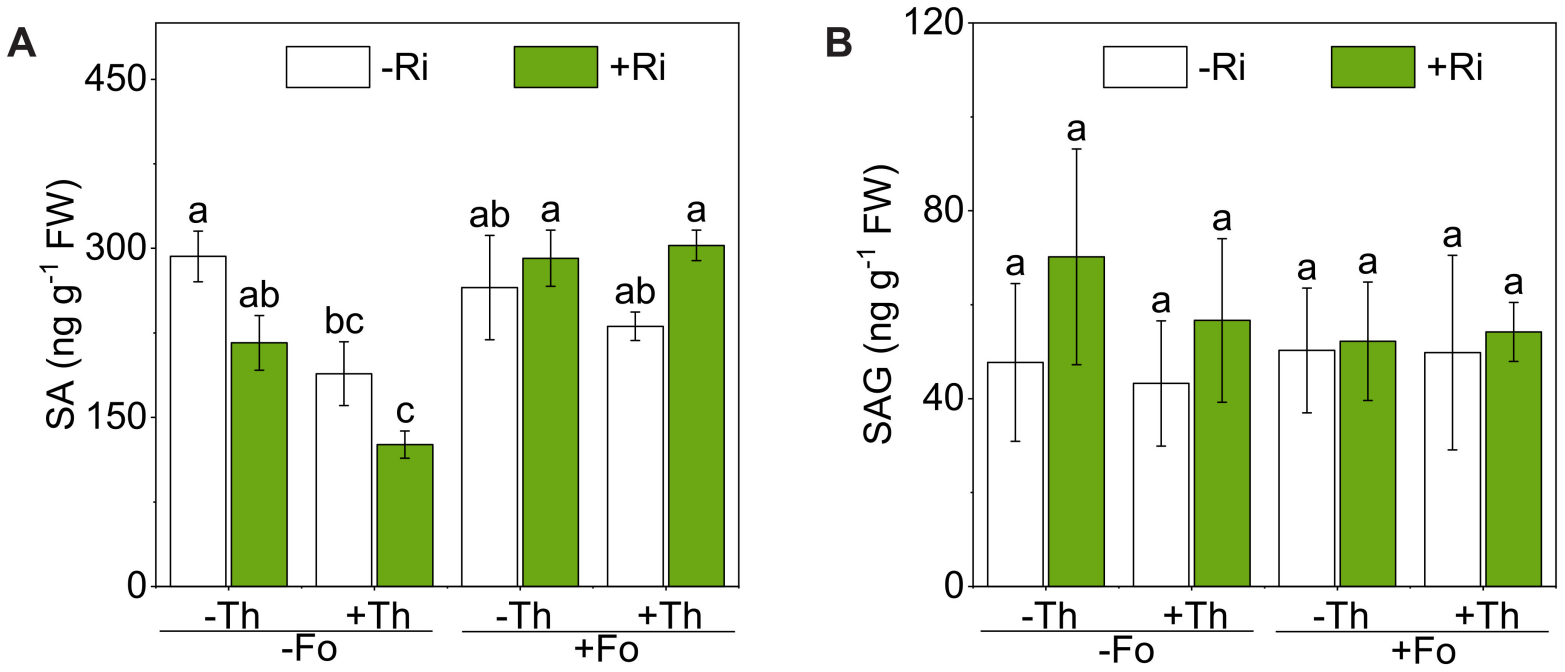
Salicylic acid (SA) **(A)** and salicylic acid 2-O-β-glucoside (SAG) **(B)** concentrations in tomato roots inoculated with/without *Rhizophagus irregularis* (Ri), *Trichoderma harzianum* (Th), and *Fusarium oxysporum* f. sp. *lycopersici* (Fo). The labels -Ri and +Ri represent non-inoculated control and inoculation with Ri, respectively. -Th and +Th represent non-inoculated control and inoculation with Th, respectively. -Fo and +Fo represent non-inoculated control and inoculation with Fo, respectively. The error bars represent the standard error (SE). Columns with the same lowercase letters indicate no significant difference at *p*<0.05.

SA accumulation was significantly modulated by Th and Fo treatments, with significant interactions detected between Myc × Fus and Tri × Fus (F(1, 15) = 10.35, *p* < 0.01; F(1, 15) = 5.40, *p* < 0.05; **Figure 2A**; **Table 1**). Notably, co-inoculation with Ri and Th followed by Fo challenge resulted in a 140.3% increase in SA concentration compared with the non-inoculated control. However, SAG did not differ among treatments (**Figure 2B**; **Table 1**).

**Figure 3:**
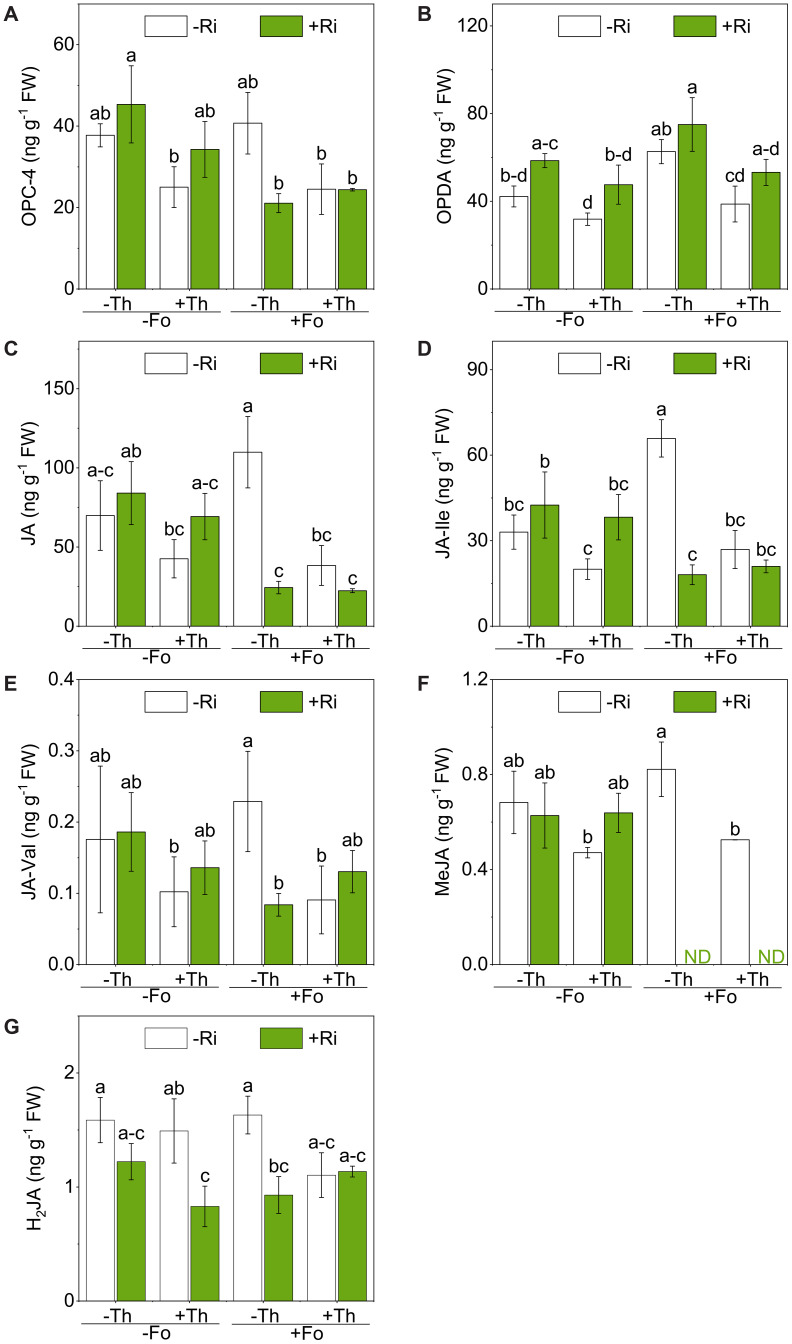
Concentrations of 3-oxo-2-(2-(Z)-pentenyl) cyclopentane-1-butyric acid (OPC-4) **(A)**, 12-oxo-phytodienoic acid (OPDA) **(B)**, jasmonic acid (JA) **(C)**, jasmonoyl-L-isoleucine (JA-Ile) **(D)**, jasmonoyl-valine (JA-Val) **(E)**, methyl jasmonate (MeJA) **(F)**, and dihydrojasmonic acid (H_2_JA) **(G)**, concentrations in tomato roots inoculated with/without *Rhizophagus irregularis* (Ri), *Trichoderma harzianum* (Th) and *Fusarium oxysporum* f. sp. *lycopersici* (Fo). The labels -Ri and +Ri represent non-inoculated control and inoculation with Ri, respectively. -Th and +Th represent non-inoculated control and inoculation with Th, respectively. -Fo and +Fo represent non-inoculated control and inoculation with Fo, respectively. The error bars represent the standard error (SE). Columns with the same lowercase letters indicate no significant difference at *p*<0.05.

Fo infection had no significant effect on OPC-4 levels (*p* = 0.072; **Figure 3A**; **Table 1**). However, this value was markedly lower in plants co-inoculated with Ri or Th, each showing significant interaction with Fo (Myc × Fus: F(1, 16) = 4.94, *p* < 0.05). Th inoculation alone had no significant effect on OPC-4 levels compared to the control. The lowest OPC-4 accumulation was observed under Ri+Fo treatment.

Fo infection significantly increased OPDA accumulation, indicating activation of the oxylipin-mediated jasmonate biosynthesis pathway (**Figure 3B**; **Table 1**). Both Ri and Th inoculation independently reduced this pathogen-induced OPDA surge, with Th exerting the strongest suppressive effect. Co-inoculation of Ri and Th further attenuated OPDA levels.

JA concentrations were not significantly affected by Fo inoculation (*p* = 0.127; **Figure 3C**; **Table 1**). Notably, Ri colonization alone did not alter JA levels, but significantly attenuated Fo-induced JA increases, as evidenced by a significant Myc × Fus interaction (F(1, 16) = 10.51, *p* < 0.01). The lowest JA concentrations were observed in plants co-inoculated with Ri, Th, and Fo.

JA-Ile was significantly induced by Fo infection (*p* < 0.05; **Figure 3D**; **Table 1**). However, this accumulation was attenuated in the presence of Ri, with significant Myc × Fus interactions (F(1, 16) = 18.90, *p* < 0.001). Th inoculation alone significantly suppressed JA-Ile levels, while Ri alone had no effect. Notably, Ri inoculation under pathogen stress resulted in the lowest JA-Ile concentrations among all treatments.

JA-Val accumulation was significantly suppressed by Th (*p* < 0.05; **Figure 3E**; **Table 1**), and a significant interaction between Th and Ri was observed. While Ri inoculation alone had no effect, its combination with Fo resulted in the lowest JA-Val content.

Ri inoculation significantly suppressed MeJA accumulation regardless of pathogen presence, and a strong Myc × Fus interaction was observed (F(1, 16) = 24.77, *p* < 0.001; **Figure 3F**; **Table 1**). Under Ri colonization, MeJA was below the limit of detection (LOD, 0.2 ng g^-^¹ FW) in Fo-challenged plants. Th inoculation also mitigated Fo-induced MeJA accumulation.

H_2_JA did not significantly increase in response to Fo infection alone (**Figure 3G**, **Table 1**). Ri significantly suppressed H_2_JA accumulation (*p* < 0.01), and a strong interaction with Fo was observed. Th inoculation showed no effect on H_2_JA levels alone but, when combined with Ri and/or Fo, further reduced H_2_JA content. The lowest H_2_JA levels were detected under the inoculation of Ri and Th.

### 3.3 Gene expression and enzyme activity

**Figure 4:**
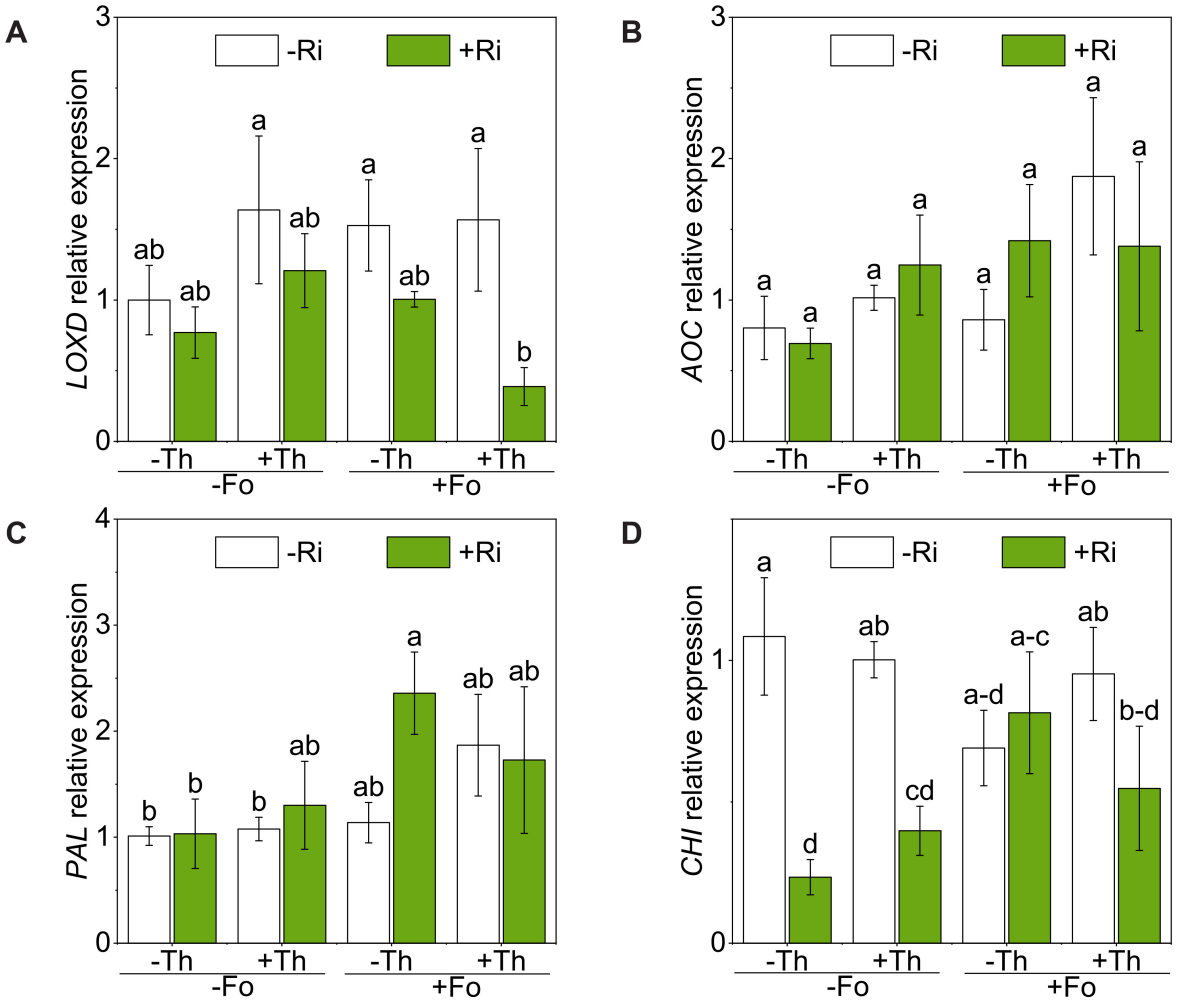
Gene expression of genes *lipooxygenase* (*LOXD*) **(A)**, ainocinnamate oxotransferase (*AOC*) **(B)**, phenylalanine ammonia lyase (*PAL)***(C)** and chitinase (*CHI*) **(D)** in tomato root inoculated with/without *Rhizophagus irregularis* (Ri), *Trichoderma harzianum* (Th), and *Fusarium oxysporum* f. sp. *lycopersici* (Fo). The labels -Ri and +Ri represent non-inoculated control and inoculation with Ri, respectively. -Th and +Th represent non-inoculated control and inoculation with Th, respectively. -Fo and +Fo represent non-inoculated control and inoculation with Fo, respectively. The error bars represent the standard error (SE). Columns with the same lowercase letters indicate no significant difference at *p*<0.05.

The expression of *LOXD*, an upstream lipoxygenase involved in JA biosynthesis, was significantly downregulated by Ri inoculation (*p* < 0.05; **Figure 4A**; **Table 1**). In contrast, *AOC* showed no significant changes across treatments (**Figure 4B**; **Table 1**).

Inoculation with Fo significantly upregulated the expression of *PAL* (*p* < 0.05; **Figure 4C**; **Table 1**). Co-inoculation with Ri and Fo further enhanced *PAL* expression by 128.3% compared with Ri alone, indicating a stronger induction under pathogen challenge. In contrast, *CHI* was significantly suppressed by Ri inoculation (*p* < 0.05; **Figure 4D**; **Table 1**). A significant Ri × Fo interaction was observed (F(1, 23) = 6.50, *p* < 0.05), where Fo infection restored *CHI* expression in Ri-colonized plants, resulting in a 2.5-fold increase compared with the Ri-alone.

**Figure 5:**
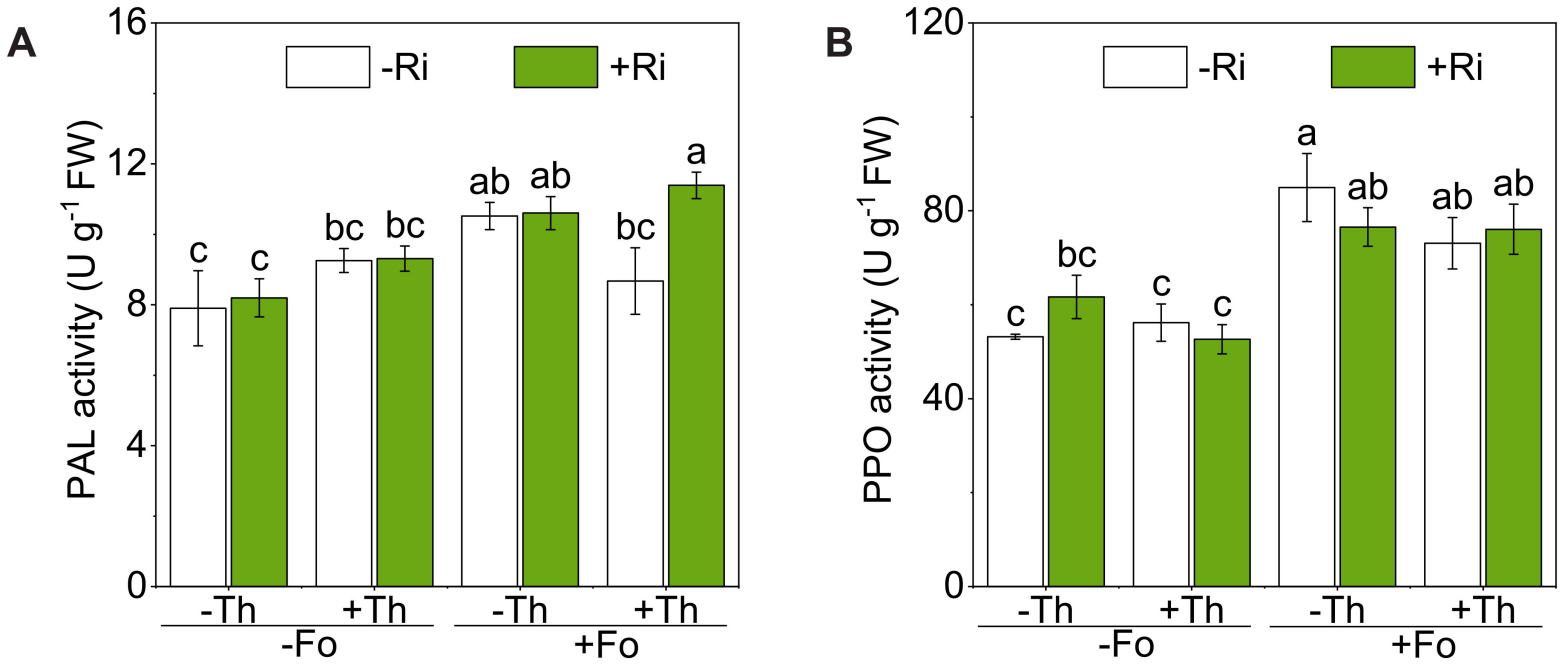
The enzyme activities of phenylalanine ammonia-lyase (PAL) **(A)** and polyphenol oxidase (PPO) **(B)** in tomato root inoculated with/without *Rhizophagus irregularis* (Ri), *Trichoderma harzianum* (Th), and *Fusarium oxysporum* f. sp. *lycopersici* (Fo). The labels -Ri and +Ri represent non-inoculated control and inoculation with Ri, respectively. -Th and +Th represent non-inoculated control and inoculation with Th, respectively. -Fo and +Fo represent non-inoculated control and inoculation with Fo, respectively. The error bars represent the standard error (SE). Columns with the same lowercase letters indicate no significant difference at *p*<0.05.

Fo inoculation significantly increased the activities of both PAL and PPO (p < 0.01; **Figures 5A, B**; **Table 1**). Ri or Th alone did not alter PAL activity compared with controls. Combining Ri or Th with Fo (Ri + Fo or Th + Fo) partially attenuated Fo-induced PAL activation, and the triple treatment (Ri + Th + Fo) showed the lowest PAL activity among Fo-challenged groups (**Figure 5A**). Neither Ri nor Th alone significantly altered PPO activity (**Figure 5B**; **Table 1**). Co-inoculation with Ri or Th attenuated the pathogen-induced increase in PPO activity, and the triple treatment (Ri + Th + Fo) exhibited significantly lower PPO activity compared with Fo alone (*p* < 0.05).

### 3.4 Correlations between *F. oxysporum* abundance and root immune parameters

**Figure 6:**
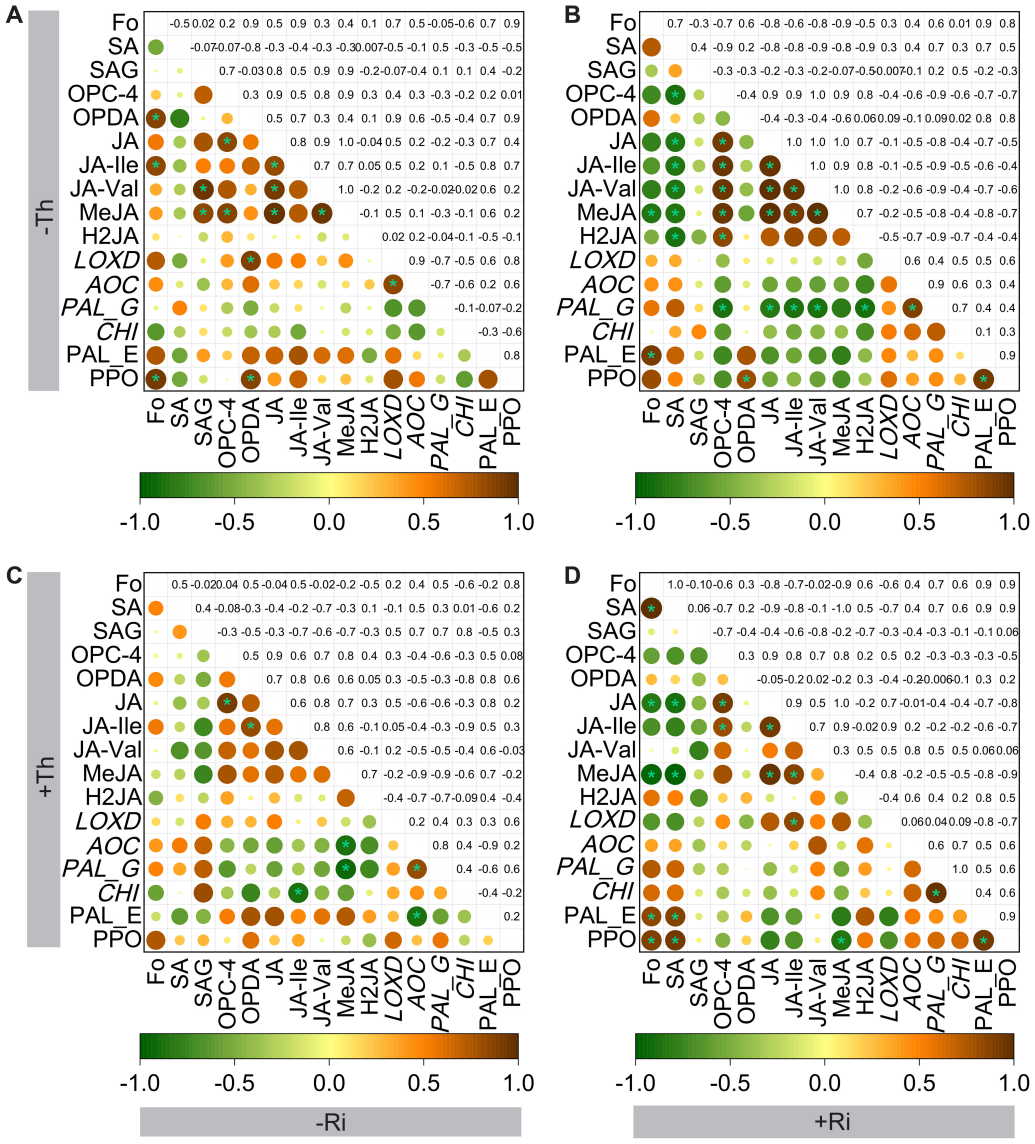
Correlation between the relative abundance of *Fusarium oxysporum*, phytohormone concentrations, gene expression, and enzyme activities in tomato roots inoculated with *Fusarium oxysporum* (Fo), with or without *Rhizophagus irregularis* (Ri) and/or *Trichoderma harzianum* (Th). Panels **(A)** show data for plants inoculated with Fo alone, without Ri and Th; **(B)** show data for plants inoculated with Fo and Ri; **(C)** show data for plants inoculated with Fo and Th; **(D)** show data for plants inoculated with Fo, Ri, and Th. An asterisk (“*”) indicates significant differences between two factors (*p*<0.05) based on the Spearman test. Pairwise comparisons of factors are represented with a color gradient denoting the Spearman correlation coefficient, where a value of 1 represents a perfect positive correlation (shown in brown), and -1 represents a perfect negative correlation (shown in green). Fo represents the relative abundance of *Fusarium oxysporum*, while SA (salicylic acid), SAG (salicylic acid 2-O-β-glucoside), OPDA (12-oxo-phytodienoic acid), OPC-4 (3-oxo-2-(2-(Z)-pentenyl) cyclopentane-1-butyric acid), JA (jasmonic acid), JA-Ile (jasmonoyl-L-isoleucine), MeJA (methyl jasmonate), H2JA (dihydrojasmonic acid), and JA-Val (jasmonoyl-valine) refer to phytohormone concentrations. *PAL_G* (phenylalanine ammonia lyase), *LOXD* (lipooxygenase), *AOC* (aminocinnamate oxotransferase), and *CHI* (chitinase) represent the relative expression levels of *PAL*, *LOXD*, *AOC*, and *CHI* genes. PAL_E and PPO represent the enzyme activities of PAL and PPO.

In plants inoculated with Fo alone (**Figure 6A**), the relative abundance of Fo showed strong positive correlations (*p* < 0.05) with OPDA (r = 0.88), JA-Ile (r = 0.85), and PPO activity (r = 0.94).

In the presence of Ri alone, the correlation between Fo abundance and immune parameters shifted (**Figure 6B**). Fo abundance was positively correlated with PAL activity (r = 0.91, *p* < 0.05) but negatively with MeJA (r = -0.86, *p* < 0.05). A negative correlation was observed between SA and several JA-related compounds (*p* < 0.05), including OPC-4 (r = −0.87), JA (r = −0.84), JA-Ile (r = −0.85), MeJA (r = −0.84), H_2_JA (r = −0.86), and JA-Val (r = −0.86).

In the Th monoinoculation treatment, no significant correlations were detected between Fo abundance and hormone concentrations (**Figure 6C**).

In the co-inoculation treatment with *Ri* and *Th* (**Figure 6D**), Fo abundance exhibited a significant positive correlation with SA concentration (r = 0.99, *p* < 0.05), PAL activity (r = 0.85, *p* < 0.05), and PPO activity (r = 0.90, *p* < 0.05), while showing a significant negative correlation with JA (r = −0.83, *p* < 0.05) and MeJA (r = −0.94, *p* < 0.05) concentrations. SA concentration was positively correlated with both PAL (r = 0.87, *p* < 0.05) and PPO activities (r = 0.93, *p* < 0.05), while negatively correlated with JA (r = −0.89, *p* < 0.05) and MeJA (r = −0.98, *p* < 0.05).

## 4 Discussion

**Figure 7:**
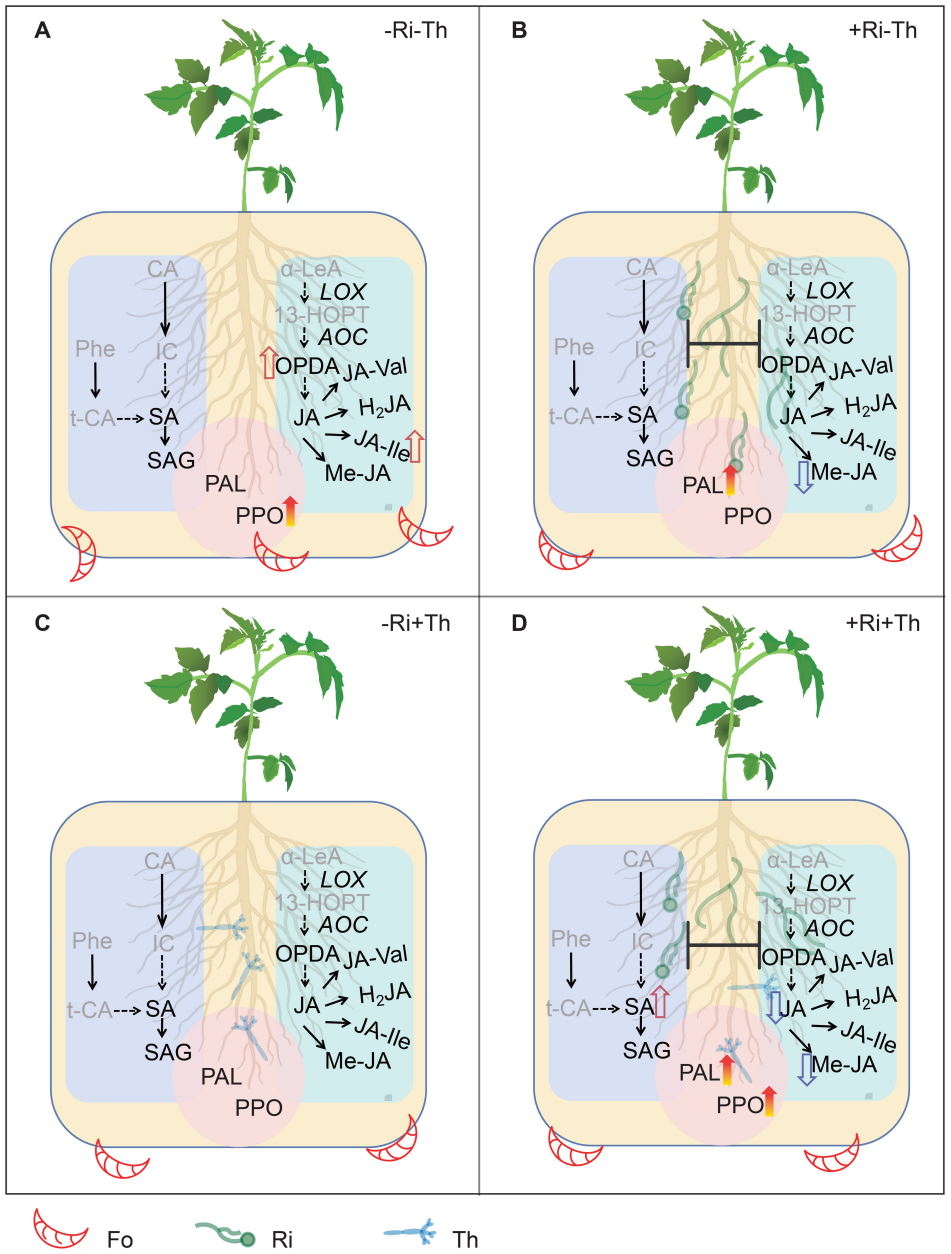
Schematic summary of salicylic acid (SA)- and jasmonic acid (JA)-mediated immune responses in tomato roots infected by *Fusarium oxysporum* f. sp. *lycopersici* (Fo), and their modulation by *Rhizophagus irregularis* (Ri) and *Trichoderma harzianum* (Th). **(A)** Plants without Ri or Th inoculation: Fo infection predominantly activated the JA signaling pathway (red thick arrows) and induced oxidative defenses through phenylalanine ammonia-lyase (PAL) and polyphenol oxidase (PPO). **(B)** Ri-inoculated plants: SA signaling was strongly enhanced, whereas JA signaling was suppressed (blue thick arrows), reflecting the antagonistic interaction between the two pathways under pathogen stress. **(C)** Th-inoculated plants: JA signaling was likewise suppressed, accompanied by a moderate promotion of SA signaling. **(D)** Ri + Th co-inoculated plants: both enhanced SA signaling and suppressed JA signaling were observed, but no synergistic effect compared with single inoculations was detected. CA, chorismic acid; IC, isochorismic acid; Phe, phenylalanine; t-CA, trans-cinnamic acid; SA, salicylic acid; SAG, salicylic acid 2-O-β-glucoside; α-LeA, α-linolenic acid; OPDA, 12-oxo-phytodienoic acid; JA, jasmonic acid; MeJA, methyl jasmonate; JA-Ile, jasmonoyl-L-isoleucine; 13-HOPT, 13(S)-hydroperoxy-octadecatrienoic acid; JA-Val, jasmonoyl-valine; H_2_JA, dihydrojasmonic acid; LOX, lipoxygenase; AOC, allene oxide cyclase; PAL, phenylalanine ammonia-lyase; PPO, polyphenol oxidase.

This study explores the complex interactions between the beneficial microbes AM fungi, *Trichoderma*, and the fungal pathogen in tomato plants. Across treatments, Ri and Th each reduced Fo abundance in roots and were associated with a shift from a jasmonate-biased profile under Fo alone to a salicylic acid-dominant state, as indicated by higher SA and PAL expression/activity together with attenuated JA-pathway readouts (OPDA, JA, JA-Ile, MeJA). Under co-inoculation, Fo suppression and SA-linked outputs were not greater than under single inoculants, supporting non-additive effects rather than functional incompatibility under our conditions (**Figure 7**).

### 4.1 Beneficial microbes mitigate *Fusarium* wilt via distinct mechanisms

The suppression of Fo colonization by beneficial microbes represents an effective and ecologically sustainable strategy for managing tomato wilt disease (Martinez-Medina et al., 2009). In this study, both Ri and Th significantly reduced Fo colonization in tomato roots (**Figure 1A**; **Table 1**). Importantly, the magnitude of suppression was comparable between the two inoculants, and co-inoculation did not lead to synergistic effects beyond single inoculations. These findings highlight non-additive rather than antagonistic outcomes under our conditions, underscoring the need to dissect the distinct but partially overlapping contributions of AM fungi and *Trichoderma* when applied together. Such mechanistic understanding is essential for the rational design of microbial consortia in integrated disease management programs.

Ri is well recognized for improving plant nutrient acquisition, particularly P, which is fundamental for plant vigor, growth, and resilience to stress (Wang et al., 2022). In our experiment, Ri inoculation markedly increased P concentrations in both shoots and roots (**Figures 1E, F**; **Table 1**) and enhanced photosynthetic performance in the absence of Fo (**Figure 1B**; **Table 1**). For instance, Pn increased by 31.1% under Ri alone, while Ri+Th co-inoculation further elevated Pn by 67.9% compared with the control. This additive benefit under pathogen-free conditions is consistent with earlier studies in crops such as cantaloupe and chickpea, where AM fungi reduced *Fusarium* wilt incidence by improving nutrient uptake and host metabolism (Singh et al., 2010). Enhanced P availability likely strengthens multiple physiological processes—including ATP synthesis, nucleic acid metabolism, and membrane integrity—that indirectly reinforce the plant’s capacity to withstand pathogen invasion (Dissanayaka et al., 2021). It is therefore plausible that Ri indirectly enhances systemic resistance by sustaining plant metabolic homeostasis and energy supply during pathogen challenge.

In contrast, Th is primarily associated with direct antagonism against soil-borne pathogens (Mukherjee et al., 2022). As a fast-growing, non-pathogenic filamentous fungus, Th rapidly colonizes the rhizosphere and root surfaces, where it suppresses pathogen proliferation through multiple mechanisms, including nutrient and space competition, hyphal parasitism, and secretion of antifungal metabolites such as peptaibols, polyketides, and volatile organic compounds (Benítez et al., 2004; Mukherjee et al., 2022). In our study, Th inoculation consistently reduced Fo colonization in tomato roots (**Figure 1A**), in line with its well-documented antagonistic properties. Previous dual-culture assays have further confirmed that Th interferes with Fo hyphal growth via mechanical penetration, coiling, and spatial exclusion (Bunbury-Blanchette and Walker, 2019; Ghanbarzadeh et al., 2014). Such evidence suggests that the reductions observed in planta can be at least partly attributed to these antagonistic mechanisms, which likely operate alongside microbe-induced changes in the rhizosphere environment.

Although Ri and Th rely on different primary routes—Ri through nutritional enhancement and Th through direct antagonism—their functional outcomes partly converge, resulting in similar reductions of Fo colonization. The absence of synergy under co-inoculation suggests that their contributions are not additive. Non-additive effects are frequently observed in AM fungi and Trichoderma studies. For example, co-inoculation of AM fungi and Trichoderma did not result in synergistic gains in controlling Fusarium wilt in banana (Castillo et al., 2019) nor in promoting onion growth (Metwally and Al-Amri, 2020). Several explanations may account for this non-additivity. First, both microbes may ultimately influence overlapping physiological processes in the host, thereby reaching a functional ceiling in terms of pathogen suppression (Martinez-Medina et al., 2016). Second, resource and niche competition between Ri and Th in the rhizosphere could constrain their combined efficacy, limiting colonization density or activity of one partner in the presence of the other (De Jaeger et al., 2011; Liu et al., 2021; Thakur et al., 2019). Third, the timing of colonization may be critical: AM fungi typically require several weeks to establish intraradical structures such as arbuscules (Shi et al., 2025; Sugiura et al., 2020), whereas *Trichoderma* colonizes root surfaces within days (Benítez et al., 2004). This temporal complementarity may allow Th to confer rapid, short-term protection, while Ri gradually builds longer-term benefits through improved host nutrition. However, when both are applied simultaneously, their overlapping downstream impacts may not translate into additive disease suppression (Martinez-Medina et al., 2011) From an applied perspective, these findings underscore the complexity of combining beneficial microbes in crop protection strategies. While both Ri and Th individually suppressed Fo, their co-inoculation did not produce additional benefits in terms of pathogen control under our experimental conditions. This suggests that designing effective consortia requires not only selecting complementary partners but also considering inoculation sequence, colonization dynamics, and the specific environmental context.

### 4.2 Hormonal signaling trade-offs underlie immune response modulation

A central outcome of this study is that inoculation with Ri or Th reshaped tomato immune responses to Fo through shifts in SA) and JA signaling. SA and JA are well established as antagonistic regulators of plant immunity (Hou and Tsuda, 2022; Pieterse et al., 2009). Our data show that Fo infection in the absence of beneficial microbes activated a canonical JA-biased response, whereas Ri or Th inoculation shifted immunity towards an SA-dominant state (**Figures 2**–**6**). This indicates that beneficial microbes do not simply enhance basal resistance but actively reconfigure hormonal priorities in the host (Pieterse et al., 2009).

In pathogen-only treatments, Fo infection strongly induced JA biosynthesis and its downstream derivatives, including OPDA, JA-Ile, and MeJA (**Figures 3**–**5**). This pattern aligns with the known association of JA signaling with defenses against necrotrophic and hemibiotrophic pathogens (Fujikawa et al., 2021; Lorenzo and Solano, 2005). Moreover, the observed positive correlations between Fo abundance and the levels of both JA-Ile and PPO activity (**Figure 6A**) are consistent with the established model that tomato plants activate JA-mediated defense pathways in response to pathogen challenge (Fernandes and Ghag, 2022).

In contrast, Ri colonization redirected the immune profile towards SA. Specifically, Ri suppressed *LOXD* expression, reduced OPDA and JA levels, and simultaneously elevated SA content and PAL expression/activity (**Figures 2A**, **3**, **4A**, **5A**). Strong negative correlations between SA and multiple JA derivatives (**Figure 6B**) reinforce the notion of SA–JA antagonism. These observations are consistent with previous reports that AM fungi can prime SA-related pathways while dampening JA signaling (Cameron et al., 2013; Hao et al., 2012). Functionally, this shift may favor resistance to the early biotrophic phase of Fo infection, when host tissue remains alive. Our results, demonstrating that Ri inoculation elevates tissue P levels and primes SA-mediated pathways, align with the broader framework proposed by Liu et al. (2024), which suggests that AM fungi modulate biotrophic pathogen defense through a balance between nutritional and defense-induced mechanisms.

Th inoculation produced a remarkably similar hormonal pattern. In Fo-challenged plants, Th reduced JA and JA-Ile accumulation while elevating SA and PAL activity (**Figures 2**–**5**). This is notable because *Trichoderma* spp. are often linked to JA/ET-dependent ISR, yet our findings and others (Harman et al., 2004) demonstrate that they can also trigger SAR-like responses mediated by SA. Such flexibility may explain their broad-spectrum biocontrol efficacy, integrating direct antagonism in the rhizosphere with systemic immune priming in the host (Alkooranee et al., 2017).

Under dual inoculation with Ri and Th, JA suppression and SA elevation were again observed, but no additive effects beyond single inoculants were detected. This plateau suggests that SA responses may reach a functional ceiling, where further enhancement is constrained by homeostatic feedbacks or growth–defense trade-offs (Li et al., 2019). Thus, the lack of synergy at the hormonal level mirrors the non-additive pathogen suppression observed at the whole-plant scale (Martinez-Medina et al., 2016).

The ecological significance of this SA–JA trade-off becomes apparent when considering the life cycle of Fo (Benjamin et al., 2022). As a hemibiotrophic pathogen, Fo begins with a biotrophic phase followed by a necrotrophic stage (Di Pietro et al., 2003). SA signaling is generally more effective against biotrophs, whereas JA defenses are essential against necrotrophs and herbivores (Grant and Lamb, 2006; Pieterse et al., 2009). By shifting tomato immunity towards SA dominance, Ri and Th may restrict Fo colonization during the biotrophic phase, thereby limiting progression to necrotrophy and effectively “buying time” for the host (Boamah et al., 2023; Zhang et al., 2013).

The link between hormones and defense enzymes further supports this interpretation. Both PAL and PPO activities increased in Ri- or Th-treated roots (**Figures 5A, B**). These enzymes reinforce cell walls through lignin deposition, accumulate phenolic compounds, and contribute to oxidative defense, thereby creating multiple barriers to pathogen invasion (Kampatsikas et al., 2019). Positive correlations between SA, PAL/PPO activity, and reduced Fo abundance (**Figures 6B–D**) suggest that these enzymatic defenses are functionally associated with the SA-biased immune state observed in treatments involving beneficial microbes.

Taken together, these findings emphasize that Ri and Th do not simply mitigate *Fusarium* wilt by reducing pathogen load but also reshape host hormonal networks. By attenuating JA signaling and reinforcing SA-dependent pathways, they help the plant reorient its immune responses in a way that is particularly effective against the early stages of hemibiotrophic infection. This microbe-driven hormonal reprogramming highlights the capacity of beneficial root symbionts to fine-tune host immunity, offering insights into both the ecological significance of symbiotic interactions and the practical potential of microbial inoculants for sustainable disease management.

### 4.3 Limitations and prospects for application

#### 4.3.1 Nutritional and immune pathways underlying disease resistance

Our findings indicate that Ri and Th alleviate Fo infection by enhancing phosphorus uptake and shifting tomato immunity toward a SA-dominated profile, leading to non-additive outcomes under co-inoculation. Both phosphorus acquisition and SA-mediated defense are key determinants of plant resistance to biotrophic pathogens (Liu et al., 2024). However, the relative contributions of nutritional and immune factors to disease suppression remain unclear.

Future studies should aim to disentangle these effects by incorporating phosphorus-matched controls, phosphorus availability gradients, and multi-timepoint sampling following pathogen challenge. These experiments will help determine whether enhanced resistance arises primarily from improved phosphorus nutrition, immune reprogramming, or their interaction, thereby elucidating nutrient–immunity crosstalk in the biocontrol process.

#### 4.3.2 Mechanistic basis of non-additive biocontrol effects

The lack of additive biocontrol effects observed under co-inoculation may result from several potential mechanisms, including niche competition between Ri and Th, partial overlap in signaling pathways regulating the SA–JA balance, or functional saturation of host defense responses (Pieterse et al., 2009). Although quantitative analysis of inoculant abundance was not performed in this study, morphological observations and successful re-isolation of both microorganisms confirmed their effective establishment in the rhizosphere. It should be noted that the present study focused on the endpoint responses of tomato plants to inoculation with Ri and Th. This design allowed for assessing the steady-state immune and hormonal adjustments occurring at the late infection stage. Nevertheless, future time-course experiments including early and intermediate stages would be highly valuable to elucidate the temporal dynamics of SA–JA pathway activation and disease suppression mechanisms.

While the present work verified the colonization of both Ri and Th, their spatial organization within root tissues was not visualized. Applying confocal microscopy coupled with fluorescent staining or *in situ* hybridization will allow high-resolution observation of colonization patterns, enabling differentiation between overlapping and spatially distinct infection sites. Understanding these spatial relationships could yield crucial insight into how AM fungi and *Trichoderma* interact functionally within the root microenvironment (Gordon, 2017). Future studies should also employ 3D root imaging or dual fluorescent labeling to clarify whether the observed non-additive effects are associated with niche sharing or spatial partitioning between Ri and Th.

#### 4.3.3 Biochemical and metabolite-level mechanisms

Although this study quantified *F. oxysporum* colonization using *pgx4*-based qPCR, which provides a reliable measure of pathogen abundance within tomato roots, this approach does not directly capture the biochemical dynamics underlying disease suppression. It is likely that part of the inhibitory effect observed under *Ri* and *Th* inoculations involves the modulation of pathogen-derived fusaric acid and the secretion of antifungal or signaling metabolites by the beneficial microbes.

Previous studies have demonstrated that *T. harzianum* produces peptaibols, polyketides, and volatile organic compounds capable of detoxifying fusaric acid and restricting *Fusarium* proliferation (Vinale et al., 2008). Moreover, AM fungi colonization can alter root exudate composition, potentially influencing rhizosphere metabolite exchange and microbial communication (Trivedi et al., 2020). Therefore, integrating targeted metabolomics with fusaric acid quantification will be valuable for elucidating how biochemical interactions among *F. oxysporum*, *R. irregularis*, and *T. harzianum* contribute to the non-additive and hormone-associated biocontrol mechanisms revealed in this study.

The *pgx4*-based quantification used in this study provides an accurate molecular proxy for pathogen load but does not directly indicate the spatial colonization or viability of *F. oxysporum* within roots. Therefore, our interpretation of reduced pathogen abundance primarily reflects changes in total fungal DNA rather than direct visualization of infection sites. Future studies combining fluorescent labeling, confocal microscopy, or histochemical staining will be valuable to confirm whether the reduced *pgx4* signal corresponds to decreased viable colonization and to elucidate how AM fungi and *Trichoderma* influence the spatial distribution of *Fusarium* within the root cortex and vasculature.

#### 4.3.4 Field validation and microbiome-based evaluation

Because the present study was conducted under controlled greenhouse conditions, the effects of soil physicochemical properties, management practices, and indigenous microbial communities remain unexplored. To translate these findings into agricultural practice, field trials should be conducted across diverse soil types and cropping systems (Martinez-Medina et al., 2016). In these trials, incorporating microbiome profiling techniques such as amplicon sequencing and metagenomic analysis will allow assessment of inoculation efficacy, community-level impacts, and the stability of disease suppression achieved by AM fungi–*Trichoderma* consortia. Such integrated analyses will provide ecological evidence for developing robust and sustainable biocontrol applications against *Fusarium* wilt.

## 5 Conclusion

This study demonstrates that both AM fungi and *Trichoderma* mitigate *F. oxysporum* infection in tomato through distinct mechanisms coupled with immune modulation. Ri or Th shifted root signaling from the JA-biased state observed under Fo alone toward an SA-biased profile, accompanied by PAL activation and attenuation of JA-pathway readouts. Co-inoculation did not enhance pathogen suppression or SA-linked outputs beyond single inoculants, indicating non-additive effect. To advance application, we recommend time-resolved and P-matched experiments to disentangle nutritional and signaling contributions, quantitative assessment of both inoculant loads and spatial niches, and factorial tests of inoculation order and formulation parameters. Field validation across soil types and management systems is essential to assess robustness within native microbiomes. Finally, integrative omics together with targeted marker assays will help resolve the circuitry of microbe-induced immune modulation and guide the rational design of microbial consortia for sustainable management of *Fusarium* wilt.

## Data Availability

The raw data supporting the conclusions of this article will be made available by the authors, without undue reservation.
